# Patient involvement in the development of clinical practice guidelines in Germany—A meta‐research study

**DOI:** 10.1002/gin2.70016

**Published:** 2025-01-28

**Authors:** Stefanie Pfisterer‐Heise, Clara Orduhan, Käthe Goossen, Jessica Breuing, Irma Hellbrecht, Sebastian von Peter, Corinna Schaefer, Dawid Pieper

**Affiliations:** ^1^ Center for Health Services Research Brandenburg Institute for Health Services and Health System Research, Faculty of Health Sciences Brandenburg Rüdersdorf Germany; ^2^ Department for Evidence Based Health Services Research, Department of Medicine Institute for Research in Operative Medicine (IFOM), Faculty of Health Herdecke University Witten Germany; ^3^ Department of Psychiatry and Psychotherapy Immanuel Klinik Rüdersdorf, Brandenburg Medical School Theodor Fontane Rüdersdorf Germany; ^4^ German Agency for Quality in Medicine Berlin Germany

**Keywords:** clinical practice guideline, consultation, Germany, participation, patient involvement

## Abstract

**Introduction:**

Patient involvement (PI) is a key element of clinical practice guidelines (CPGs). However, PI in CPGs often falls short of quality standards. For evidence‐ and consensus‐based CPGs in Germany, a study in 2018 showed that a mere 58% had included a patient (participation), 56% with voting right. We aimed to provide an update on whether and how patients were involved in CPGs (participation and consultation), on the availability of patient versions (PVGs) (communication) and to investigate whether CPGs in Germany adhere to the RIGHT checklist.

**Methods:**

An electronic search on the Register of the Association of Scientific Medical Societies was performed. Eligibility criteria were defined a priori. We included evidence‐ and consensus‐based CPGs valid on 31 March 2023. A data extraction form including 35 items was designed and piloted. Five researchers independently extracted data from CPGs, methodology reports and PVGs. Data were analysed descriptively.

**Results:**

The search identified 135 CPGs meeting the eligibility criteria. Participation has increased to 79% (107/135 CPGs), in 73% of CPGs (98/135) with voting right. Participation was more common in CPGs for chronic than acute conditions (76/86 CPGs, 88% chronic vs. 23/38 CPGs, 61% acute). Consultation was scarcely employed (3/135 CPGs, 2%). Communication, operationalised as PVGs being available on the internet, increased from 33% (35/105) in 2018 to 43% (58/135) in 2023. 26% of CPGs (28/107) with participation reported on patients' selection as required by the RIGHT checklist.

**Discussion:**

PI has improved, although around 20% of CPGs were still developed without participation. This applies in particular to CPGs on acute conditions where PI should be strengthened. At the same time, innovative and efficient methods for consultation and for evaluating patients' impact are required. Moreover, communication should be expanded. To further improve transparency in CPGs, guideline development groups should specifically report on patients' recruitment and selection.

## Key points

*What Is already known on this topic?*

- Patient involvement is a key element of clinical practice guidelines (CPGs).
- For evidence‐ and consensus‐based CPGs in Germany, a study in 2018 showed that a mere 58% had included a patient (participation), 56% with voting right. Patient versions were available on the internet for 33% of CPGs (communication).

*What this study adds?*

- We aimed to provide an update on whether and how patients were involved in CPGs (participation and consultation), on the availability of patient versions (communication) and to investigate whether CPGs in Germany adhere to the RIGHT checklist.
- Participation increased to 79%, in 73% of CPGs with voting right. It was more common in CPGs for chronic than acute conditions (88% vs. 61%). In contrast, consultation was scarcely employed (2%). Communication increased to 43%.
- 26% of CPGs with participation reported on patients' selection as required by the RIGHT checklist.

## 1 INTRODUCTION

Patient involvement (PI) is internationally acknowledged as a key element of trustworthy evidence‐based clinical practice guidelines (CPGs).^1^, ^2^, ^3^, ^4^, ^5^ The Guidelines International Network (GIN) distinguishes between three PI strategies: Consultation is defined as collecting information from patients, for example, by means of surveys, focus groups or literature searches.^1^ Participation means the exchange of information between guideline developers and patients, often implemented through patient representation in guideline development groups. Communication encompasses the dissemination of information to patients, for example, through the production of patient versions of CPGs (PVGs).^1^

As consultation, participation and communication strategies entail different characteristics, they are applied in different stages of the CPG development process.^1^, ^6^ For instance, consultation methods can help to gather the views of a large number of individuals.^1^ Therefore, they are especially suited to inform the topic of a CPG and to comment on the draft guideline.^6^ In contrast, participation methods can help to promote a shared perspective of patients and professionals. They are particularly appropriate to discuss and agree on guideline content when drafting recommendations.^1^, ^6^ Lastly, communication strategies are typically used in the dissemination and implementation stage of CPG development. They can help to support patients' individual health care decisions, for example, by fostering shared‐decision making.^1^

For advancing PI in CPG development, the international scientific community has developed helpful material. This comprises practical guidance, checklists as well as assessment and reporting tools on CPG development, all including PI.^5^, ^7^, ^8^, ^9^, ^10^, ^11^, ^12^ A specific checklist for when and how to involve stakeholders in CPG development is being worked on.^13^, ^14^ For the development of PVGs, several recommendations and a reporting checklist have been formulated.^15^, ^16^, ^17^, ^18^, ^19^ Despite this, the implementation of PI differs considerably between countries. For instance, while the National Institute for Health and Care Excellence (NICE) has always involved patients since starting its work on CPGs in 2001, in the United States, only 8% of guideline development organisations required PI in 2017.^20^ Accordingly, in 2017, a US‐American study found that only 13% of guideline developers engaged patients or the public at least some of the time.^20^

In Germany, the Association of Scientific Medical Societies (Arbeitsgemeinschaft der Wissenschaftlichen Medizinischen Fachgesellschaften [AWMF]) already since 2012 requires that representatives of the patient target population should be included in CPG development at an early stage.^21^ This rule has been mandatory in order to classify a CPG as evidence‐ and consensus‐based.^21^ Since 2020, the AWMF requires ‘depicting the perspective of patients/the affected population’ (own translation) when developing evidence‐ and consensus‐based CPGs. Wherever possible, PI should be implemented through direct involvement of patient organisations, that is, participation.^22^ However, in cases in which participation is not possible, the AWMF instead points out to consultation methods, such as literature searches on perspectives of those affected as well as surveys or focus groups.^22^

Despite PI in CPG development being mandatory for evidence‐ and consensus‐based CPGs in Germany, in 2018, a study found that a mere 58% of guideline development groups of evidence‐ and consensus‐based guidelines had implemented participation, with no data on consultation methods.^23^ Communication, operationalised as a PVG being available, was only achieved for 35% of CPGs.^23^ Overall, the authors concluded that PI in CPG development in Germany was ‘insufficient’ at the time of the study.^23^

This study is an update of the 2018 study^23^ with the following three aims: (1) to describe the current status on whether and how patients were involved in the development of CPGs in Germany (consultation and participation), (2) to provide an update on the availability of PVGs (communication) and to explore whether patients were involved in their development and (3) to investigate whether CPGs adhere to the RIGHT checklist^7^ with respect to their reporting of PI.

## 2 METHODS

Reporting of this meta‐research study follows the guidelines set forth in the Preferred Reporting Items for Systematic Reviews and Meta‐Analyses (PRISMA) 2020 Statement, where applicable (Supporting Information S1: Appendix 3).^24^ A study protocol was published a priori on protocols.io.^25^

### 2.1 Eligibility criteria

**Guideline documents:** We included information from the following three types of guideline documents in our analysis:

1. *CPGs:* As eligibility criteria for CPGs, we defined evidence‐ and consensus‐based CPGs (S3 CPGs) valid on 31 March 2023, produced by any scientific medical society in Germany, available in their long version. In contrast to other international CPG classifications, the CPG system in Germany has a hierarchical structure with the following levels of CPGs: S1 (lowest level) Recommendations by groups of experts, S2k Consensus‐based guidelines, S2e Evidence‐based guidelines and S3 Evidence‐ and consensus‐based guidelines characterised by a representative committee, systematic review and synthesis of the evidence and a structured consensus process.^26^
2. *PVGs:* The terminology and reporting with respect to PVGs vary widely.^27^ We defined PVGs as follows: All documents that were directly derived from CPGs and were recognisably directed at patients as the target group. These included short patient information leaflets and flyers that were directly derived from a CPG.
3. *Methodology reports:* Methodology reports for each specific CPG or PVG could be part of the document or be available as separate documents.

**Patient groups:** We differentiated between the following four patient groups:

1. Patients nominated by self‐help groups, as a proxy for people with lived experience.
2. Family members nominated by self‐help groups, for example parents in the case of CPGs on diseases affecting children or family members of a patient with a mental health disorder.
3. Patient advocates nominated by patient organisations not specifically indicated as self‐help groups.
4. Patients without any link to a self‐help group or patient organisation.

**PI strategies:** In accordance with the GIN public toolkit,^1^ we differentiated between the following three PI strategies:

1. Participation, that is, patient representation in the guideline development group.
2. Consultation, that is, methods to collect information from patients. As consultation methods, we included literature searches on patients' preferences, surveys, focus groups and interviews.
3. Communication, operationalised as a PVG being available.

### 2.2 Information sources and search strategies

For retrieving all relevant S3 CPGs, one researcher (S.P.‐H.) conducted an electronic search on the Register of the Association of Scientific Medical Societies in Germany (https://register.awmf.org/de/suche).

The Register was last searched on 31 March 2023 using the advanced search:

1. status ‘valid guidelines’,
2. document type ‘clinical practice guideline (long version)’,
3. development status ‘S3’,
4. society ‘All’ and
5. organisation ‘All’.

For retrieving PVGs of included S3 CPGs, three researchers (J.B., K.G. and I.H.) searched the Register of the Association of Scientific Medical Societies in Germany (https://register.awmf.org/de). For PVGs referring to cancer, the homepage overview of PVGs of the German Guideline Program in Oncology (GGPO, Leitlinienprogramm Onkologie, https://www.leitlinienprogramm-onkologie.de/patientenleitlinien/uebersicht) was searched.

### 2.3 Selection and data collection process

For identifying all CPGs meeting eligibility criteria, one researcher (S.P.‐H.) carried out the search on the Register of the AWMF and exported the titles of S3 CPGs to a table in Excel format. Two researchers (S.P.‐H. and D.P.) designed and piloted a data extraction form to extract data from eligible guideline documents. Moreover, two researchers (S.P.‐H. and C.O.) developed a codebook for coding all items (Supporting Information S1: Appendix 2). For example, we coded the condition addressed in a CPG as ‘acute’ if the CPG refers to an acute event such as stroke, deals with surgery or topics related to surgery or pain. In contrast, we coded a condition as ‘chronic’ if a disease is chronic and if a disease can be acute or chronic. As cancer has at least phases with chronic characteristics or is already described as a ‘new chronic disease’ based on the fact that many patients nowadays live with it for many years, we categorised cancer as a chronic condition.^28^, ^29^ We coded a condition as ‘other’ if the CPG refers to a topic other than a specific disease, for example, hormonal contraception. Data extraction was then tested on a set of 10% of CPGs and the codebook was further refined.

A team of two reviewers (S.P.‐H. and C.O.) worked independently to extract relevant data from S3 CPGs (long version) and their corresponding methodology reports. For S3 CPGs, the AWMF provides a template in which the topic patient and public involvement is located in the chapter on the ‘Composition of the guideline group’. Despite this template, we found that information on PI could potentially be located throughout the CPG and methodology report. We therefore decided posthoc to search all S3 CPGs and their corresponding methodology reports using key words such as ‘interviews’ (Supporting Information S1: Appendix 2).

A team of three researchers (J.B., K.G. and I.H.) independently extracted data from the PVGs as well as their corresponding methodology reports. Extracted data were compared and discrepancies were resolved through discussion. In case of discrepancies regarding information extracted from PVGs, a fourth reviewer (S.P.‐H.) helped to resolve conflicts.

### 2.4 Data items

We developed a data extraction sheet including 35 items. We included

1. all items from the 2018 study on PI, for example, patient was a member of the guideline development group, PVG available.^23^
2. Items from the RIGHT (Reporting Items for practice Guidelines in HealThcare) checklist with reference to PI in CPGs.^7^ In case an item from the RIGHT checklist contained two pieces of information, we split this item into two items (i.e., item 9a RIGHT checklist ‘Describe how all contributors to the guideline development were selected and their roles and responsibilities…’ was split into the two items: (a) Information on selection process of patient and (b) Information on roles and responsibilities of patient).
3. Further items of interest, such as items on training and payment of patients.

### 2.5 Data analysis

All data were analysed descriptively, for instance, by calculating sums and percentages. In case of items included in the 2018 study, for example, ‘Classification of leading professional society according to subject area’, we followed the analysis of the former study to allow for comparability.

## 3 RESULTS

### 3.1 General information on S3 CPGs

The search yielded 202 S3 CPGs presented as ‘valid’ in the AWMF Register. Of these, 135 S3 CPGS were in fact valid on 31 March 2023 and thus included in the analysis. Of these 135 S3 CPGs, 44 S3 CPGS had been included in the 2018 study (as identified by the register number), but in a previous version.

Of the 135 S3 CPGs included in this analysis, six CPGs (4%) were developed as part of the programme for National Disease Management Guidelines (Nationale VersorgungsLeitlinien [NVL]) dealing with highly prevalent chronic diseases such as asthma, chronic obstructive pulmonary disease (COPD) or diabetes. Within the framework of the German Guideline Program in Oncology, 25 CPGs (19%) were developed and 104 CPGs (77%) were developed outside these programmes. Of 135 CPGs, 38 (28%) were referring to acute medical conditions, 86 (64%) were referring to chronic conditions and 11 (8%) were referring to other conditions. Surgical as well as general and internal medicine societies led the development of 46 CPGs (34%) each, followed by 16 CPGs (12%) developed under the leadership of medical societies in the category ‘Nervous and emotional disorders’ (Supporting Information S1: Appendix 1).

### 3.2 PI (participation and consultation) in the development of S3 CPGs

In 2023, 134/135 CPGs (99%) reported on PI in comparison to 99/105 CPGs (94%) in 2018. Of the 135 CPGs that were included in the current analysis, 108 CPGs (80%) had involved patients, either in the form of participation or consultation. Of the 27/135 CPGs (20%) without PI, only 3/27 (11%) CPGs mentioned this as a limitation (Table 1).

**Table 1:** Information related to participation and consultation in clinical practice guidelines (CPGs) in Germany (2023).

	Yes	No
Information on whether patients' values and preferences were considered **during CPG development** a	134/135 (99%)	1/135^b^ (1%)
Patients' values and preferences were considered **during CPG development** a	108/135 (80%)	27/135 (20%)
Explanation in case patients' values and preferences were not considered **during CPG development** a	0	27/27 (100%)
Information on limitations in the process of CPG development, for example, if patients' values and preferences were not sought	3/27 (11%)	24/27 (89%)
Information on how the validity of recommendations might have been affected in case patients' values and preferences were not sought	3/27 (11%)	24/27 (89%)

a Items printed in bold modified in comparison to the RIGHT checklist.^7^

b The CPG without information on PI was further categorised as not having implemented PI.

### 3.3 Participation in the development of S3 CPGs

In 107/135 CPGs (79%), patients were involved as members of the guideline development group. In 98/135 CPGs (73%), patients had voting rights. In comparison to 2018, participation in CPG development has thus risen by 21%. Patients were more often involved in CPGs referring to chronic conditions than in CPGs referring to acute or other conditions (Table 2).

**Table 2:** Participation in clinical practice guidelines (CPG) development (2018 vs. 2023).

	2018	2023
Patient(s) participation in the guideline development group	61/105 (58%)	107/135 (79%)
Acute condition	n.c.^a^	23/38 (61%)
Chronic condition	n.c.	76/86 (88%)
Other condition	n.c.	7/11 (64%)
Patient(s) had voting right	59/105 (56%)	98/135 (73%)

a n.c. not collected.

#### 3.3.1 Guideline development group and numbers of patient members

On average, there were 33 members in the guideline development group (min 6, max 118). In 55/107 CPGs (53%), only one patient was involved. There were at most 6 patients in a guideline development group (min 1, max 6, mean 1,5).

#### 3.3.2 Patients' selection, roles and background

We found only limited information with respect to patients' selection. Specifically, only 28/107 CPGs (26%) reported on patients' selection, for example, that a search for suitable patient organisations had been carried out and/or that several organisations had been contacted and only the participating organisation had agreed to send a delegate.

#### 3.3.3 Training and payment for patients

Only one CPG reported on a training on the methodology of evidence appraisal. This training was, however, aimed at the whole guideline development group and not specifically at patients. Just one CPG reported on patients' reimbursement, more specifically on refund of travel expenses (Tables 3 and 4).

**Table 3:** Information on participation, that is, patient(s) being (a) member(s) of the guideline development group (2023).

	Yes	No	Unclear
Information on patient(s) being a member of the guideline development group	134/135 (99%)	1/135 (1%)	0
Patient(s) was/were (a) member(s) of the guideline development group	107/135 (79%)	28/135 (21%)	0
Information on patients' name(s)	105/107 (98%)	2/107 (2%)	0
Information on the name of patient organisation(s)	101/107 (94%)	6/107 (6%)	0
Information on the selection process of patient(s)	28/107 (26%)	79/107 (74%)	0
Patient(s) had medical background	12/107 (11%)	10/107 (9%)	85/107 (79%)
Prior training for patient(s)	1/107 (1%)	1/107 (1%)	105/107 (98%)
Information on patient's role(s) and responsibilities (e.g., steering group, guideline panel, etc.)	103/107 (96%)	4/107 (4%)	0
Information on whether patient(s) had voting right	99/107 (93%)	8/107 (7%)	0
Patient(s) had voting right	98/107 (92%)	1/107 (1%)	8/107 (7%)
Patient(s) exercised voting right	93/107 (87%)	5/107 (5%)	8/107 (8%)
Payment/reimbursement for patient(s)	1/107 (1%)	70/107 (65%)	36/107 (34%)

*Note*: Percentages might not add up to 100% due to rounding.

**Table 4:** Frequency of patient group status (2023).

Patient group status	Number of clinical practice guidelines
Patients nominated by self‐help groups only	41/135 (30%)
Patient advocates only	22/135 (16%)
Family members nominated by self‐help groups only	6/135 (4%)
Patients only	3/135 (2%)
Patients nominated by self‐help groups and patient advocates	8/135 (6%)
Patients and family members nominated by self‐help groups	3/135 (2%)
Patient advocate(s) and family members nominated by self‐help groups	1/135 (1%)
Patients nominated by self‐help groups and patient advocates and family members nominated by self‐help groups	1/135 (1%)
Unclear	22/135 (16%)
Not applicable	28/135 (21%)

### 3.4 Consultation in the development of S3 CPGs

Consultation methods were applied in the development of 3/135 CPGs (2%). Of these, only one CPG (1%) reported on exclusively using a consultation method, namely, a literature search on patients' views and preferences. The use of participation and consultation methods was reported by 2/135 CPGs (1%). More specifically, one CPG reported on implementing participation and a patient survey regarding patient‐relevant outcomes, while the other CPG reported on participation and hearings as well as an online survey among treatment‐seekers and treatment‐experienced people.

### 3.5 Communication: PVGs of S3 CPGs

For 74/135 CPGs (55%), a PVG was planned according to the methodology report and/or CPG (long version). For 58/135 PVGs (43%), at least one PVG was available on the internet. Compared to 2018, more PVGs were available on the internet: 35/105 PVGs (33%) in 2018 versus 58/135 PVGs (43%) in 2023. Participation in PVG development groups slightly declined, with 33/40 PVGs (83%) in 2018 versus 46/58 PVGs (79%) in 2023 (Supporting Information S1: Appendix 1).

## 4 DISCUSSION

Although PI is internationally acknowledged as a quality criterion in the development of CPGs, its implementation is not yet comprehensive. In Germany, participation in the development of CPGs has increased from 58% in 2018 to 79% in 2023. Also, with 73%, more patients had the right to vote in 2023 compared to 56% in 2018. However, several implementation challenges of PI in CPG development remain.

Still approximately 20% of S3 CPGs were developed without PI, although this is mandatory.^30^ There was more participation in CPGs on chronic conditions than in those on acute conditions. It could be hypothesised that patients with acute conditions are less organised in associations than those with chronic conditions. Therefore, guideline development groups working on a CPG on an acute condition might simply not have found a suitable patient. However, in Germany, there are so‐called key patient and consumer organisations that might have been able to establish a suitable contact.^31^

As we identified insufficient reporting on patients' recruitment and selection, we cannot rule out that guideline development groups did contact the key patient organisations and still did not find a suitable patient. On the one hand, this lack of reporting might be due to the fact that reporting according to the RIGHT checklist is not mandatory in Germany. On the other hand, a description of the selection process is not part of the AWMF S3 CPG template. Furthermore, in Germany, there is no such national coordinating organisation as NICE where patients can apply to get involved in CPG development. However, patients' selection and thereby their legitimisation are major factors for trust in CPGs. Reporting on patients' recruitment should therefore be mandatory. In the long term, a structured consented process could help to increase transparency in recruiting and selecting patients for CPG development in Germany, while making it easier for guideline development groups to find suitable patients.

Our analysis shows that patient support for taking part in guideline development groups is either not very well developed or not well reported, or both. Particularly, we found only limited information on patients' reimbursement as well as on the provision of patient trainings. Trainings are, however, essential to prepare patients for taking part in guideline development groups, not only with respect to content knowledge but also for dealing with group dynamics and potential physician resistance to lay involvement.^32^, ^33^, ^34^, ^35^ In the same way, patients should be compensated for their involvement in CPGs, first to allow poorly resourced patients to take part in CPG development and, second, to avoid the risk of them being funded by other sources with potential COI.^12^ We cannot rule out that patients were trained or were reimbursed. For instance, the provision of trainings is part of the NVL programme, but not yet part of reporting in the NVL CPGs. However, awareness for patients' needs when taking part in the development of CPGs should be reflected in providing patients with knowledge and money and reporting on it.

Currently, it is not reported how patients are actually engaged in CPG development in Germany, which is in accordance with findings from other countries such as Canada.^33^ The consistent use of frameworks for engaging patients could add value in this respect.^36^ Furthermore, the following two questions for evaluating PI in CPGs should be addressed on a regular basis: How do patients assess the process and outcomes of CPG development? What was patients' impact on CPG development? The mandatory implementation of tools such as PANELVIEW or the Patient Engagement Evaluation Tool (PEET‐12) could provide valuable insights on these issues,^37^, ^38^ while allowing for further evaluation of these instruments.

To further evaluate the impact of PI in CPG development, reporting on how patients have actually been involved in the CPG development process needs to be improved. This could be achieved by mandating defined, structured reporting on PI. As an added benefit, reporting requirements may help to overcome potential barriers such as disregard for patients’ contributions.^39^ Greater appreciation of patient input may in turn lead to patients being granted more speaking time at guideline meetings than the current level of only up to 5%.^40^

Our analyses show that only a few consultation methods are currently being used in CPG development in Germany.^41^ On the one hand, this might be due to a lack of awareness or lack of prioritisation of consultation methods among guideline developers; on the other hand, resources might be lacking. For a higher utilisation of consultation methods, these should be further promoted within the AWMF Guidance. Furthermore, publishing the results of patient surveys or interviews within CPG development should be encouraged.

While improved usage of consultation methods could be helpful in implementing PI when participation is not possible, it could also enable a broader application of PI methods. In our study, only 1% of CPGs reported using more than one method of PI. However, this is in contrast to patients' wishes who prefer the availability of different ways of PI throughout the process,^42^ specifically because in patients' opinions, ‘genuine and equal interaction’ in the guideline panel is questionable.^42^ However, to increase the application of consultation methods, guideline development groups might need innovative and efficient methods for use during guideline development. For example, in Finland, patients preferred reference groups as a participation method over participating in guideline panels as members.^42^ Other innovative procedures to provide insights regarding patients' values and preferences include guideline panel surveys.^43^, ^44^ Although these are good steps forward, it remains unclear whether these will come to the same results as compared to surveys among patients.

Finally, more than half of the CPGs (55%) indicated that a PVG was planned for the future, but the actual availability of PVGs in 2023 was only 43%. While this represents an increase compared to 2018 (33% PVGs available in 2018), there is still much room for improvement.

## 5 LIMITATIONS

There are several limitations with respect to our study: First, we did not analyse ‘whether values and preferences of the target population(s) were considered in the formulation of each recommendation’, as required by the RIGHT checklist.^7^ However, as this is currently generally not documented in CPGs in Germany, we have reformulated this item into the broader item ‘values and preferences of the target population were considered during guideline development’ and furthermore collected data on patients' voting right. As explanation for not seeking patients' values and preferences, we defined providing an explanation for not using any form of PI, which might be a rather strict criterion. Furthermore, we did not include ‘Commenting’ as a consultation method, as in our sample of CPGs, ‘Commenting’ often seemed to refer to inviting individual patients for comments. However, this does not correspond to the GIN definition of consultation of reaching large groups of patients. Lastly, we found it difficult to categorise patients into patients nominated by self‐help groups and patient advocates, as oftentimes, only very rudimentary information on patients' group status was provided.

## 6 CONCLUSION

In conclusion, PI in CPG development has risen in Germany, with more patient participation, and more patients having the right to vote. At the same time, communication, in the form of available PVGs of CPGs, has improved, making it easier for patients to access evidence‐based health information. With respect to consultation, innovative and effective methods need to be developed to facilitate reaching larger groups of people as well as collecting data from patients who are harder to involve in participation methods.^1^ Moreover, general guidance on PI in CPGs should be developed and provided in the future, as is currently underway.^13^, ^14^ In Germany, a structured consented process could help to recruit patients specifically in the case of CPGs referring to acute and other conditions as well as to support transparency in recruiting and selecting patients in general. Further research should focus on barriers to implementing PI.

## AUTHOR CONTRIBUTIONS

**Stefanie Pfisterer‐Heise**: Conceptualisation; methodology; formal analysis; investigation; data curation; writing—original draft and visualisation. **Clara Orduhan**: Conceptualisation; methodology; formal analysis; investigation; writing—review and editing. **Käthe Goossen**: Conceptualisation; methodology; formal analysis; investigation; writing—review and editing. **Jessica Breuing**: Conceptualisation; methodology; formal analysis; investigation; writing—review and editing. **Irma Hellbrecht**: Conceptualisation; methodology; formal analysis; investigation; writing—review and editing. **Sebastian von Peter**: Conceptualisation; writing—review and editing. **Corinna Schaefer**: Conceptualisation; writing–review and editing. **Dawid Pieper**: Conceptualisation; methodology; resources; writing—review and editing, supervision and project administration.

## CONFLICT OF INTEREST STATEMENT

The authors declare no conflicts of interest.

## ETHICS STATEMENT

The authors have nothing to report.

## Supporting information

Supporting information.

Supporting information.

Supporting information.

## Data Availability

The data that support the findings of this study are openly available in protocols.io protocols.io at https://www.protocols.io/joinworkspace/medical-school-brandenburg/QRFLA.
