# Supplementary material for: Patient involvement in the development of clinical practice guidelines in Germany—A meta‐research study
Source: Clin Public Health Guidel. 2025 Jan 28;2(2):e70016. doi: 10.1002/gin2.70016 (PMC13563351; doi:10.1002/gin2.70016)
Supplement: Supplementary file 1 [file GIN2-2-e70016-s001.docx]

**Appendix 1**

*Table 1 Frequency of leading professional society in CPG development according to subject area (2018 vs. 2023)*

|  | **2018** | **2023** |
| --- | --- | --- |
| Pediatric specialties | 11/105 (10%) | 13/135 (10%) |
| Surgical specialties | 31/105 (30%) | 46/135 (34%) |
| Nervous and emotional disorders | 12/105 (11%) | 16/135 (12%) |
| Infections / Environment – Prevention, Therapy | 2/105 (2%) | 7/135 (5%) |
| General and Internal Medicine | 40/105 (38%) | 46/135 (34%) |
| Oncology only – without pediatric specialties | 9/105 (9%) | 4/135 (3%) |
| Other | 0 | 3/135 (2%) |

*Table 2 Information related to PVGs (2023)*

|  | **Yes** | **No** | **Unclear** |
| --- | --- | --- | --- |
| Methodology report as separate document | 119/135  (88%) | 16/135  (12%) | 0/135  (0%) |
| PVG available or planned as stated in methodology report or long version of CPG | 74/135  (55%) | 61/135  (45%) | 0/135  (0%) |
| Availability of a PVG on the internet | 58/135  (43%) | 77/135  (57%) | 0/135  (0%) |
| Participation (all patient groups) in PVG | 46/58  (79%) | 5/58  (9%) | 7/58  (12%) |

*Table 3 Information related to PVGs (2018 vs. 2023)*

|  | **2018** | **2023** |
| --- | --- | --- |
| Methodology report as separate document | 96/105  (91%) | 119/135  (88%) |
| PVG available or planned as stated in methodology report or long version of CPG* | 50/105  (48%) | 74/135  (55%) |
| Availability of a PVG on the internet | 35/105*  (33%) | 58/135  (43%) |
| Patient participation (all patient groups) in PVG | 33/40**  (83%) | 46/58  (79%) |

* As analyzed in 2023.

** In 2018 for 35/105 CPGs at least one PVG was available (as counted in the data reported). In total 40 documents for patients were available as for 3 CPGs more than one patient document was found in 2018.
