# Supplementary material for: Patient involvement in the development of clinical practice guidelines in Germany—A meta‐research study
Source: Clin Public Health Guidel. 2025 Jan 28;2(2):e70016. doi: 10.1002/gin2.70016 (PMC13563351; doi:10.1002/gin2.70016)
Supplement: Supplementary file 2 [file GIN2-2-e70016-s002.docx]

**Appendix 2**

*Codebook*

| **Column**  **(excel-file)** | **Item** | **Description/ Annotations** | **Example** |
| --- | --- | --- | --- |
| A | Consecutive number of S3 CPGs included in the analysis |  |  |
| B | Register number | As stated in the AWMF Register (always without blank characters), all register numbers of the Guideline Program Oncology (Leitlinienprogramm Onkologie) are marked OL at the end | 021-003 (register number CPG “Pancreatitis”), 032-010OL (register number CPG “Exocrine pancreatic carcinoma”) |
| C | In 2018 analysis included | Code as “Yes” (1) in case that the S3 CPG was also included in the 2018 study according to the AWMF Register number [1]. |  |
| D | In 2018 valid until | Referring to S3 CPGs analyzed in the 2018 study. State expiry date of S3 CPG. | 27.02.2020 (expiry date of Register number 017-064) in 2018. |
| E | Name of CPG (English own translation), [original German title] | Title of CPG without supplementary information “S3-Leitlinie” (S3 medical guideline”) | “Pancreatitis” (Pankreatitis) |
| F | Version | As stated in the AWMF Register (and/or the CPG (long version)) | 3.1 (version number CPG “Pancreatitis”) |
| G | Date last edited | As stated in the AWMF Register (and/or the CPG (long version)) | 10.09.21 (date last edited CPG “Pancreatitis” |
| H | Valid until | As stated in the AWMF Register (and/or the CPG (long version)). | 30.04.26 (expiry date CPG “Pancreatitis”) |
| I | Leading professional society in CPG development | State just one professional society leading guideline development | German Society for Gastroenterology, Digestive and Metabolic Diseases (leading professional society in guideline development of S3 “Pancreatitis”) |
| J | Classification of leading professional society according to subject area | Code according to Appendix F of 2018 study [1] | Deutsche Gesellschaft für Allgemeinmedizin und Familienmedizin (German Society for General Practice and Family Medicine) classified as “General and Internal Medicine”=5 |
| K | Medical condition of CPG (acute/ chronic/other) | Code as “acute”, if CPG refers to an acute event such as polytrauma, stroke, etc., deals with surgery or topics related to surgery or pain. Code as “chronic”, if a disease is chronic (including cancer) and if a disease can be acute or chronic. Code as “Other”, if CPG refers to another topic than a specific disease. | Acute: S3 guideline “Infarct-related cardiogenic shock”;  Chronic: S3 guideline “Cystic fibrosis in children in the first two years of life”;  Other: S3 guideline “Hormonal Contraception” |
| L | Information on whether patients‘ values and preferences were considered during CPG development | Code as “Yes” (1) in case that a CPG contained any information that a method for PI was used, such as 1. Including (a) patient(s) as (a) member(s) of the guideline development group, 2. Conducting (a) patient survey(s), interviews or focus groups with patients, conducting a literature search for studies on patients’ preferences. (In accordance with the AWMF Regelwerk [2] “Consultation” was not included as a method of PI.) Code as “No” (0) in case that PI (including any methods of PI) was not mentioned. | “Yes”: CPG Anal Cancer “To assess the relevance of the endpoints considered, a survey was conducted among the members of the guideline committee and among patients with anal cancer…” (methodology report, p. 19) |
| M | Patients‘ values and preferences were considered during CPG development | Code as “Yes” (1) in case that any PI method was/ were used. For included PI methods, see above. | See above. |
| N | Explanation in case that patients’ values and preferences were not considered during CPG development | Code as “Yes” (1) in case that reasons were given, why none of the afore mentioned methods of PI (e.g. patient a member of the guideline development group, survey, literature search, etc.) was feasible. Code as “No” (=0), if for instance there was only an explanation provided why no patient could be involved. | “No”: CPG “Sore throat” “No patient representatives were included in this update, as the topic is predominantly acute illnesses and there are no national or international patient organisations on the topic of sore throat.” (methodology report, p. 6) |
| O | Information on limitations in the process of CPG development, e.g. if patients’ values and preferences were not sought | Code as “Yes” (1), if in the section on limitations or implementation of the respective CPG, the lack of PI is clearly addressed as a limitation. | “Yes”: CPG “Temporomandibular joint dislocation” Chapter 5. Interdisciplinary consensus, review and approval. "Since no specific patient organization exists on the topic of "temporomandibular joint dislocation" and no general self-help group or patient association has expressed an interest in participating, an orienting search was carried out, including Internet forums, to take the patient perspective into account, last updated 07/2022. Since this also did not yield any hits, the guideline is primarily aimed at the practitioners of temporomandibular joint dislocation." |
| P | Information on how the validity of recommendations might have been affected in case that patients’ values and preferences were not sought | Code as “Yes” (1), if it is clearly indicated, how the lack of PI might affect the validity of the CPG, for instance with respect to the user target group. | “Yes”: CPG “Temporomandibular joint dislocation” "Since no specific patient organization exists on the topic of "temporomandibular joint dislocation" and no general self-help group or patient association has expressed an interest in participating, an orienting search was carried out, including Internet forums, to take the patient perspective into account, last updated 07/2022. Since this also did not yield any hits, the guideline is primarily aimed at the practitioners of temporomandibular joint dislocation." (methodology report, p. 20) |
| Q | Information on patient(s) being (a) member(s) of CPG development group | Code as “Yes” (1) in case that a CPG contained any information that (a) patient(s) was included as (a) member(s) of the guideline development group. Code as “No” (0) in case that patient participation was not mentioned. | “Yes”: CPG “Gastric cancer” “The CPG was developed with the direct involvement of a patient representative, Mrs Barbara Kade.” (CPG long version, p. 14). |
| R | Patient(s) was/were (a) member(s) of CPG development group in 2023 (all CPGs) | Code as “Yes” (1) in case that a (a) patient(s) in 2023 was/ were member(s) of the CPG development group. Code as “No” (0) in case that (a) patient(s) was/were only invited for proofreading or consultation. | “Yes”: CPG “Gastric cancer” “The CPG was developed with the direct involvement of a patient representative, Mrs Barbara Kade.” (long version, p. 14). “No”: CPG “Treatment of acute perioperative and post-traumatic pain” “The organization Schmerzlos e.V. was actively involved in the consultation phase” (methodology report, p. 8) |
| S | Number of members in CPG development group in the current version of CPG | Count all members of CPG development group for current version of CPG except moderators, methodological consulting and deputies. |  |
| T | Number of patients in CPG development group in the current version of CPG | Count patient members. Do not count deputies except it was explicitly reported that deputies were (a) member(s) of a working group. |  |
| U | Information on patient’s name(s) | Code as “Yes” (1) in case that patient’s name(s) was/ were given. | “Yes”: CPG “Gastric cancer” “The guideline was developed with the direct involvement of a patient representative, Mrs Barbara Kade.” (long version, p. 14). “No”: CPG “Eating disorders” “In the absence of a Germany-wide self-help group, three former patients were invited to the final consensus conference as patient spokespersons (without giving their names) in order to ensure the direct involvement of patients.” (methodology report, p. 3) |
| V | Information on name of patient organization(s) | Code as “Yes” (1) in case that name of patient organization was given. As patient organizations we particularly defined self-help groups plus the so-called key patient organizations in Germany, such as for example Deutsche Arbeitsgemeinschaft Selbsthilfegruppen (German Working Group for Self-Help Groups). We did not for instance include the Bundeszentrale für gesundheitliche Aufklärung (BZgA, Federal Centre for Health Education) as a patient organization/ organization of those affected, as the BZgA is aiming to fulfil prevention and health promotion tasks for the federal government, i.e. their task is educating the public, not speaking for them. | “Yes”: CPG “Diagnosis, treatment and aftercare of laryngeal carcinoma”. Bundesverband der Kehlkopfoperierten e.V. (Federal Association of Laryngectomees e.V.) |
| W | Name of patient organization | Free text. |  |
| X | Information on selection process of patient(s) | Code as “Yes“ (1) in case that it was described how the patient(s) was/ were recruited, for instance which patient organizations were contacted and/ or that a a specific search was carried out, which patient organizations are in the field and/ or why a particular patient organziation was selected. | “Yes”: CPG “Back pain in children and adolescents”, “The CPG was created in the consensus phase with the direct involvement of patient representatives. Mrs Heike Norda, chairwoman of the association SchmerzLOS e.V., was entitled to vote and was involved in the creation of the guideline. Her participation is representative of affected children and adolescents, and the previously intensive search for children and adolescents from the patient group to participate in the guideline development group from 2018 – 2020 was unsuccessful. Some of the underage patients approached did not have the consent of either their parents or the children themselves. Attempts to ask former adolescent – now adult – patients to participate in the guideline development group were also unsuccessful…” (CPG, long version, p. 101). |
| Y | Patient(s) had medical background | Code as “Yes“ (1) in case that a patient or one of the patients (if several were members of the guideline development group) was a physician, as indicated by the title “Dr. med”. Besides screening all CPG long versions and guideline reports, both aforementioned categories of documents were searched using the patient’s last name(s) as keywords. | “Yes”, CPG “Diagnostics, therapy and aftercare for patients with follicular lymphoma”, Holtkamp, Ulrike, Dr. med. (CPG long version, p. 320) |
| Z | Prior training for patient(s) | Code as “Yes“ (1) in case that it was reported that the patient(s) received any kind of training. Code as “Unclear” (2), if no information on any kind of training for patients was reported. Besides screening all CPG long versions and methodology reports, both aforementioned categories of documents were searched for the following keywords: “*Training*“ (training), “*Schulung*“ (training/ instruction), “*Vorbereit**“ (preparat*). | “Yes”, CPG “Neurological rehabilitation for coma and severe impairment of consciousness in adulthood”, “Before the start of the critical evidence appraisal, the members of the guideline development group were trained twice on the methodology of evidence appraisal.” (methodology report, p. 19) |
| AA | Information on patient’s role(s) and responsibilities, (e.g. steerig group, guideline panel, etc.) | Code as “Yes“ (1) in case that it was reported that the patient(s) was/ were (a) member(s) of the consensus group or a specific working group or in case that it was reported that the patient(s) was involved in all steps of guideline development. Code as “No” (0), if it was reported that the patient(s) was/ were involved in guideline development. | “Yes”: CPG “Gastric cancer” “The guideline was developed with the direct involvement of a patient representative, Mrs Barbara Kade. She had an equal function as a mandate holder. She was particularly active in working group 7.” (CPG long version, p. 14). |
| AB | Information on whether patient(s) had voting right | Code as “Yes“ (1) in case that a CPG contained any information on patient’s voting right. | “Yes”: CPG “Screening, diagnosis and treatment of alcohol-related disorders”, “Mrs Barbara Hansen was involved in the update as a representative of the self-help association “Guttempler in Deutschland” on behalf of the patients/ representatives. However, she had no voting right in this capacity.” (methodology report, p. 6) |
| AC | Patient(s) had voting right | Code as “Yes“ (1) in case that it was reported that the patient(s) had voting right. | “Yes”: CPG “Gastric cancer” “The guideline was developed with the direct involvement of a patient representative, Mrs Barbara Kade. She had an equal function as a mandate holder.” (CPG long version, p. 14). |
| AD | Patient(s) exercised voting right | Code as “Yes” (1) in case patient(s) had no COI. Code as “No” a patient COI was reported or if patient(s) opted not to vote. | “No”: CPG “Prevention, diagnosis, therapy and aftercare of lung cancer” Baysal, Barbara. Topics affected by COI: ALK, Immune therapy (moderate). Abstention from voting.” (methodology report, p. 345) |
| AE | Payment/ reimbursement for patient(s) | Code as “Yes“ (1) in case that it was reported that the patient(s) received any kind of payment or reimbursement. Code as “Unclear” (2), if no information on any kind of payment/ reimbursement for patients was reported. Besides screening all CPG long versions and methodology reports, both aforementioned categories of documents were searched for the following keywords: “Finanzierung“ (funding), “ehrenamtlich“ (honorary/ voluntary). | “Yes“: CPG “Borderline Personality Disorder”: “The funds were used to finance the personnel costs for the scientific coordination and editing of the guideline, to finance the independent moderation of the guideline meetings by the AWMF moderators, the travel costs of the AWMF moderators and the representatives of those affected (borderline trialogue) and for the event costs during the face-to-face-meetings.” (CPG long version, p. 109) |
| AF | Patients’ group status (Patient(s) mandated by self-help groups only/ Patient advocate(s) only/ Family member(s) mandated by self-help groups only/ Patient(s) only/ Patient(s) mandated by self-help groups & patient advocate(s) / Patient(s) & family member(s), both mandated by self-help groups, Patient advocate(s) & family member(s) mandated by self-help groups, Patient(s) mandated by self-help groups & patient advocate(s) & family member(s) mandated by self-help groups | Code as “Patient(s) mandated by self-help group“, in case that from the name of the patient organization it can be concluded that the patient organization is a self-help group. Code as “Patient advocate“, in case that from the name of the patient organization it can concluded that the patient is employed as patient advocate or in case that in the COI section the patient organization is stated as the patient’s employer. Code as “Family member(s) mandated by self-help group“, in case that from the name of the patient organization it can be concluded that the organization is an organization for patient’s relatives or in case that the degree of relationship is given, for example “patient’s father”. Code as “patient” in case that the patient is referred to as individual patient without naming any organization. Code as “Unclear” in case that one or all patients’ status are unclear. | “Patient mandated by self-help group”: CPG “Prevention, diagnosis, therapy and aftercare of lung cancer”, Name of patient organization: BSL Bundesverband Selbsthilfe Lungenkrebs e.V. (Federal Association for Lung Cancer Self-Help), “Patient Advocate”: CPG “Analgesia, sedation and delirium management in intensive care medicine”, Name of patient organization: “Patientenbeauftragte für das Land Berlin” (Patient advocate for the federal state of Berlin), “Family member”: CPG “Compulsive disorders”, Name of patient organization: Bundesverband der Angehörigen psychisch erkrankter Menschen (BApK) e.V. (Federal Association of relatives of mentally ill people) |
| AG | Consultation methods to identify patients‘ values and preferences | Besides screening all CPG long versions and methodology reports, both aforementioned categories of documents were searched using the following keywords: “Survey”, “Interview”, “Focus group”, “Literature search”. Literature search was only included in case that it explicitly referred to capturing patients’ perspectives. | Further method “Literature search”: CPG “Acute and chronic cough” “In order to capture the perspective of patients with acute and chronic cough, a selective literature search on acute cough and a systematic search on the patient perspective of people with chronic cough were conducted.” (methodology report, p. 7) |
| AH | Methodology report | Methodology report defined as a separate document describing the methods used for CPG development. Code as “Separate document” (1) or “Included in long version of CPG” (0). In case a separate document is limited to the evidence synthesis, code as “Included in the long version of CPG” (0). |  |
| AI | In 2023 PVG available or planned as stated in methodology report or long version of CPG | Code as “Yes” (1) if patient document is available or planned according to CPG long version or methodology report (in contrast to the 2018 study which only included patient documents mentioned in the methodology reports). |  |
| AJ | In 2023 PVG available on the internet (all CPGs) | Code as “Yes” (1) in case that in 2023 PVG was available. Search for patient documents on the webpage of the CPG (AWMF Register) and the organisation responsible for producing oncological guidelines (“Leitlinienprogramm Onkologie”). |  |
| AK | PI in PVG | Code as “Yes” (1) if at least one author of the PVG is identified as a patient or member of a patient organization, or identifiable as such by cross-checking with the participant list in the guideline document, “No” (0) if a patient is clearly not involved, “Unclear” (9) if insufficient information is provided, and “Not applicable” (8) if no PV of the CPG was found. |  |
| AL | Editorial coordination | Provide name of organization responsible for editorial coordination (other than the guideline-producing professional society), or code as “Unclear” (9), if insufficient information is provided, and “Not applicable” (8) if no PVG of the CPG was found. | Leitlinienprogramm Onkologie, ÄZQ |
| AM | Patient(s) was/were (a) member(s) of CPG development group in 2018  (CPGs 2018 and 2023 only) | Code as “Yes” (1) in case that the 2018 analysis stated that (a) patient(s) was/were a member(s) of CPG development group, referring only to those CPGs included in 2018 and 2023 analysis. |  |
| AN | Patient was a member of CPG development group in 2023 (CPGs 2018 and 2023 only) | Code as “Yes” (1) in case that (a) patient(s) in 2023 was/ were a member(s) of the CPG development group, referring only to those CPGs included in 2018 and 2023 analysis. |  |
| AO | PVG available on the internet in 2018(CPGs 2018 and 2023 only) | Code as “Yes” (1) in case that the 2018 analysis stated that PVG was available, referring only to those CPGs included in 2018 and 2023 analysis. |  |
| AP | PVG available on the internet in 2023 (CPGs 2018 and 2023 only) | Code as “Yes” in case that in 2023 PVG was available, referring only to those CPGs included in 2018 and 2023 analysis. |  |
|  | Homepage of PVG (only included in 2018 analysis) | Data not retrieved. |  |

1.	Ollenschlager, G., et al., *[Patient involvement in clinical practice guidelines is poor after 12 years of German guideline standards: A review of guideline methodologies].* Z Evid Fortbild Qual Gesundhwes, 2018. **135-136**: p. 50-55.

2.	Arbeitsgemeinschaft der Wissenschaftlicher Medizinischer Fachgesellschaften e.V. *AWMF Regelwerk*. 2023 [cited 2023 23.11.2023]; 2.1:[Available from: <https://www.awmf.org/regelwerk/zusammensetzung-der-leitliniengruppe-beteiligung-von-interessengruppen>.
